# The Overlap Between Crohn’s Disease and Intestinal Tuberculosis: A Never-Ending Story

**DOI:** 10.3390/medicina62040794

**Published:** 2026-04-21

**Authors:** Sergiu Marian Cazacu, Costin Teodor Streba, Cristian Constantin, Claudiu Marinel Ionele, Ion Rogoveanu, Alexandru Valentin Popescu, Mirela-Marinela Florescu

**Affiliations:** 1 Gastroenterology Department, University of Medicine and Pharmacy Craiova, 200349 Craiova, Romania; sergiu.cazacu@umfcv.ro (S.M.C.); ion.rogoveanu@umfcv.ro (I.R.); 2 Clinical Emergency Hospital Craiova, 200642 Craiova, Romania; costin.streba@umfcv.ro (C.T.S.); mirela.florescu@umfcv.ro (M.-M.F.); 3 Pneumology Department, University of Medicine and Pharmacy Craiova, 200349 Craiova, Romania; 4 Imaging Department, University of Medicine and Pharmacy Craiova, 200349 Craiova, Romania; 5 Doctoral School, University of Medicine and Pharmacy Craiova, 200349 Craiova, Romania; popescualexandruvalentin97@yahoo.com; 6 Gastroenterology Department, Clinical Emergency Clinical Hospital, 200239 Craiova, Romania; 7 Pathology Department, University of Medicine and Pharmacy Craiova, 200349 Craiova, Romania

**Keywords:** Crohn’s disease, intestinal tuberculosis, granuloma

## Abstract

The prevalence of Crohn’s disease has increased over the last few decades, even in developing countries, whereas that of intestinal tuberculosis has decreased, which places both diseases at an epidemiological crossroads. Crohn’s disease and intestinal tuberculosis share many clinical, endoscopic, imaging, and pathological features, which sometimes make differential diagnosis very difficult; an accurate diagnosis is, however, very important since an erroneous treatment can worsen the evolution or delay proper therapy. The association between past TB infection and Crohn’s disease can make the diagnosis especially hard. This review summarizes current data on specific features that allow differentiation between Crohn’s disease and intestinal tuberculosis, paying particular attention to the microbiome, clinical signs, endoscopy, cross-sectional imaging, bacteriological, and immunological findings detailed. The importance of computerized models and scores for the differentiation is also detailed, because common features may make the differentiation based on a single criterion difficult.

## 1. Introduction

The incidence of pulmonary and extrapulmonary tuberculosis has markedly decreased in Western countries [1]; in contrast, in Asia and Africa, the prevalence is still significant [2], with HIV infection being one of the important factors of resurgence in some countries [3,4]. *Mycobacterium tuberculosis* establishes a lifelong infection, and approximately one quarter of the world’s population carries this organism, with a risk of progressing to active disease [5]. Despite being preventable and treatable, tuberculosis still affects about 10 million new people and causes over a million deaths each year. Although most cases involve the lungs, about 15–20% cases in immunocompetent persons worldwide are extrapulmonary TB [3,6]. Among these, abdominal tuberculosis is a common form, typically involving the gastrointestinal tract, peritoneum, lymph nodes, and occasionally solid organs [7]. Intestinal tuberculosis (ITB) is the sixth site of extrapulmonary TB, noted in 11% of all TB patients [8,9], and the ileocecal area is the preferred location in 90% of cases [9,10]. A pan-European study conducted between 2002 and 2011 observed a declining incidence of pulmonary TB, with a stationary incidence of extrapulmonary forms; gastrointestinal TB is the eighth most common extrapulmonary location, and positive cultures are noted in only 30.3% of cases [11].

One of the most difficult differentiations in ITB is represented by Crohn’s disease (CD). CD incidence is increasing worldwide, although stabilization was observed during the last decade, which may imply an epidemiological crossroad between CD and ITB in developing countries [1,5,12,13]. In India, although the prevalence of ITB is significant [14], the CD burden is also high, in second place worldwide, after the US [3]; a colonoscopic study of more than 30,000 patients with lower gastrointestinal symptoms in a large population area has noted 992 cases of CD and 364 ITBs, which implies that even in a country with a high rate of tuberculosis, such as India, CD diagnosis outnumbers ITB cases [15]. However, other studies conducted in India have not shown a significant change in trend in ITB or CD diagnosis over 2005–2019 [16]. An increasing trend in the diagnosis of CD and a decreasing trend in the diagnosis of ITB were also noted in Korea [17]. Other diseases, such as ischemic or chronic non-specific enteritis and Behcet disease, may also mimic ITB [13].

We performed a comprehensive review of current data regarding the differentiation between ITB and CD. We searched PubMed, Web of Science, Google Scholar, and Scopus databases by using the following search terms: “Intestinal tuberculosis” OR “Gastrointestinal tuberculosis” AND “Crohn disease”. All relevant articles were manually evaluated, and those suitable for analysis were retained. Articles were grouped into comprehensive and rapid reviews, systematic reviews and meta-analyses, and original studies (epidemiology, microbiome, proteomics, endoscopic, cross-sectional, pathology, IGRA + nucleic acid testing, bacteriology, and multiparametric and computerized models). Detailed data regarding original articles analysed for the differentiation between ITB and CD are found in Supplementary File S1: Table original studies.

## 2. Epidemiological Trend and Misdiagnosis Rates Between CD and ITB

Clinical, imaging, and laboratory data in CD and ITB are very often similar, and many patients with CD are initially diagnosed with ITB in endemic countries [18,19]. The misdiagnosis rate was not negligible, with 11% ITB cases diagnosed as CD and 18% of CD as ITB in a South Korean study [12], which also noted a declining temporal trend of misdiagnosing CD as ITB but an increasing trend of misdiagnosing ITB as CD [12]; another study performed in China found that 65% CD cases were initially diagnosed as ITB [18]. In Europe, a study of 47 patients diagnosed with CD in Portugal found that only two cases were reclassified as ITB during reevaluation, which represents 4.3% [20]. The misdiagnosis of inflammatory bowel disease (IBD) can delay appropriate therapy and contribute to poorer clinical outcomes even after the correct diagnosis is established. Some patients with CD may have a previous latent TB infection, which can further complicate the diagnosis. In cases of suspected IBD—particularly when treatment response is suboptimal—clinicians should systematically evaluate chest imaging and pathological samples for ITB suggestive features to ensure accurate differentiation between the two conditions [21].

The overlap between clinical, imaging, and pathological features appear in 60–70% of the cases [22]; high misdiagnosis rates are primarily attributed to the low sensitivity of the methods used for ITB definitive diagnosis, ranging from 5.3% to 37.5% for the fast-acid bacilli technique, 23% to 46% for culture, and 36.4% to 67.9% for PCR [23]. Extrapulmonary TB cases are often paucibacillary, yield low microbiological confirmation, and may mimic many other conditions. Clinicians can rely on non-specific indicators, such as ascitic fluid adenosine deaminase in patients with peritoneal TB; when uncertainty persists, especially in endemic areas, an antitubercular therapeutic trial can be used [6]. As the burden is highest in low-resource settings, the development of standardized, evidence-based diagnostic and treatment approaches has progressed more slowly [24].

## 3. Microbiome, Immunology, and Proteomic Particularities

**Figure 1:**
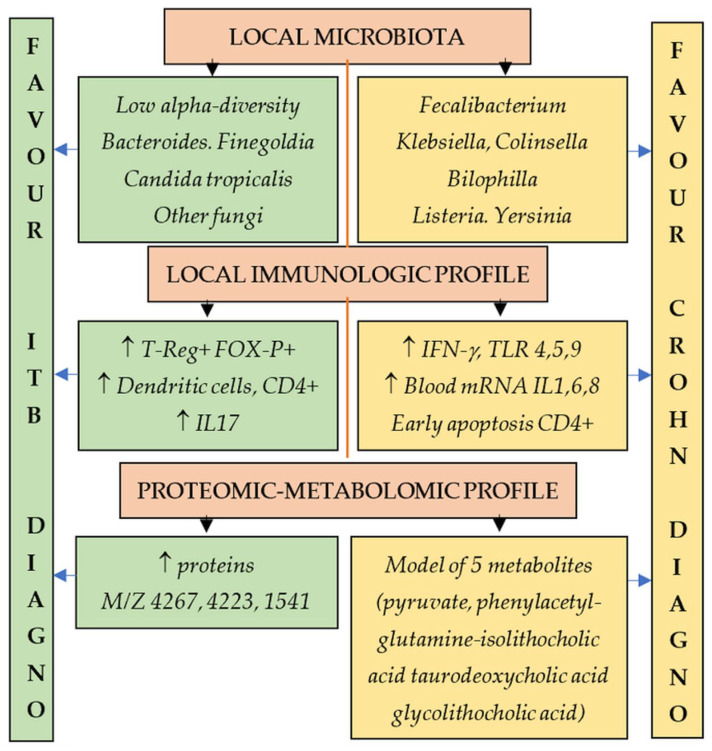
Microbiota, immune, proteomic, and metabolomic profiles in ITB and CD. ↑ = increased presence.

Although both diseases share common pathogenic mechanisms, such as local inflammation, the factors that initiate and perpetuate inflammation and subsequent complications, such as genetic predisposition, diet, childhood nutritional status, and exposure to antibiotics, oral contraceptives, NSAIDs, and immunological abnormalities, are different between the two diseases, which may help to differentiate between the two [3,25]. Both ITB and CD may have specific epigenetic, immunologic, proteomic, and metabolomic signatures, or alterations in the gut microbiome (Figure 1), which can be used for differentiation [26].

Several proteomics-based approaches for signatures specific to CD and ITB have been made [27,28,29,30,31,32]; a diagnostic model based on serum proteomic fingerprints identified three discriminatory peaks at M/Z 4267, 4223, and 1541, the latter being identified as LOLX2, that may help to differentiate between CD and ITB [31]. A panel of five serum metabolomic biomarkers evaluated by targeted liquid chromatography–mass spectrometry (pyruvate, phenylacetylglutamine, isolithocholic acid, taurodeoxycholic acid, and glycolithocholic acid) was also helpful for the differentiation in a study, with an AUC (Area under ROC Curve) of 0.963 [33]. Clinical application remains unclear and difficult to replicate.

Immune profiling may aid in the differentiation [34,35,36,37,38,39,40,41]. A combination of plasma microRNA-375-3p with Eotaxin-1/CCL11, SDF-1α /CXCL12, and G-CSF has demonstrated an AUC of 0.83 for discriminating between ITB and CD [36]. The level of regulatory FOX-P3+-T reg cells was lower in CD and higher in ITB in initial and validation studies [34,35,37]. Peroxisome proliferators in PPAR-gamma pattern, IL4/5/6, and Toll-like receptor pathways were significantly downregulated in CD [34]; the classical complement pathway was upregulated, and FOXP3 mRNA was significantly overexpressed in colonic tissue in ITB patients [34]. A higher proportion of dendritic cells (CD56^-^ CD68- CD163^-^ CD11c^+^ HLA-DR^+^) and CD4+ cells and a lower proportion of early apoptotic CD4+ cells were noted in ITB; in contrast, the proportion of dendritic cells was lowest in CD [38,41]. The predictive role of anti-I2 antibodies (anti-Pseudomonas fluorescens-associated sequence I2) in differentiating ITB from CD was analyzed in a study, in which a moderate value of AUC was noted [42]. The cytokine profile may be different in ITB compared to CD; a study found that in intestinal biopsies the expression of growth-related oncogene-alpha was increased in ITB, and interferon-gamma and TLR 4, 5, and 9 expressions were increased in Crohn’s disease, while in peripheral blood mononuclear cells the mRNA expression of IL-1, IL-6, and IL-8 mRNA was upregulated in CD and IL-17 upregulated in ITB [43]. The lack of availability of the methods used, the lack of confirmation in other studies, and the small number of patients significantly limit the clinical applications.

Gut microbiota characteristics and the presence of pathobionts may differ between patients with ITB and CD. A limited number of studies are available; a higher proportion of *Bacteroides* and *Escherichia coli* was recorded in ITB patients compared with CD [44,45], with lower alpha diversity [45,46]. A higher proportion of *Bacteroides*, *Faecalibacterium*, *Collinsella*, and *Klebsiella* was also found in CD, and the four-bacterial-strain-based model demonstrated an AUC of 0.976 [45]. A study in India has found increased fungal diversity in both ITB and CD, and divergent mycobiome between the diseases, with *Bifidobacterium*, *Finegoldia*, *Candida tropicalis*, *Alternaria metachromatica*, and *Phanerochaete* specifically enriched in ITB and *Bilophila* enriched in CD but not in ITB [46]. A study of pathobionts has shown a higher prevalence of *Listeria monocytogenes* and *Yersinia enterocolitica* in patients with CD compared to ITB [47].

## 4. Clinical Pattern in CD and ITB

CD is more indolent, whereas ITB has a shorter onset (6–7 months) [3,14,48]. Some clinical features, such as abdominal pain, diarrhea, and weight loss, are encountered in both diseases; fever and night sweats are more common in ITB, especially in the evening, and less common in uncomplicated CD [3,8,49,50]. Severe weight loss, ascites, abdominal lumps, and pulmonary symptoms (cough and hemoptysis) are suggestive of ITB, whereas perianal involvement, hematochezia, and extraintestinal manifestations are more commonly observed in CD [3,5,8,12,14,51]. It is worth noting that only 20–25% of ITB had concomitant pulmonary disease [3,48]. Rectal involvement is more common in CD, although some cases of rectal ITB were also reported [52,53]. In a Bayesian meta-analysis by Limsrivilai, chronic diarrhea, hematochezia, and perianal signs were more common in CD, whereas fever and night sweats were more common in ITB [54]. In a small study comparing 26 CD and 26 ITB patients, a history of hematochezia, diarrhea, longer symptom duration, and the absence of fever predicted CD with an 85% accuracy [51]. In a study by Liu et al., rural patients presenting with abdominal pain, transverse ulcers, or caseating granulomas were more often diagnosed with ITB, whereas urban patients more commonly demonstrated altered stool patterns, significant anemia, bowel wall thickening, rectal involvement, skip lesions, and irregular glandular architecture, features favoring CD [55]. Among younger individuals (<30 years), systemic symptoms, abdominal masses, perforation, and the need for emergency surgery were more characteristic of ITB [55]. In another study published by Larsson et al., ITB patients tended to have a lower BMI (Body mass index) than those with CD; however, overt cachexia was reported equally in both groups. Symptom duration before presentation was similar, and abdominal pain with altered bowel habits commonly occurred across both conditions, with comparable bowel frequency. Only ITB patients reported chest symptoms, which were absent in CD [56]. Some studies reported a high frequency of extraintestinal TB, with 41.2% in another location [10].

Differentiating between ITB and CD may be particularly important in certain patient categories. In the pediatric population, the differentiation between CD and ITB is especially critical as misdiagnosis can lead to lifelong growth retardation or unnecessary exposure to toxic antitubercular therapy (ATT) [57]. Research indicates that in children, ITB is more likely to present with systemic symptoms, abdominal masses, and a higher frequency of emergency complications such as perforation. Conversely, bloody stools and left-sided colonic involvement are stronger independent predictors of CD in this age group in multivariate analysis [58].

Pregnancy represents another unique diagnostic challenge, as physiological changes can mask or mimic TB symptoms. Active TB during pregnancy is associated with a ninefold increase in the risk of antenatal admission and miscarriage. Furthermore, maternal TB poses a severe risk to the fetus, with a sixfold increase in perinatal deaths and a high risk of vertical transmission. If left untreated, TB in pregnancy leads to poor obstetric outcomes, including low birth weight and preterm labor [59].

In elderly patients or those who are significantly malnourished, the clinical presentation of ITB may be even more deceptive. These patients often exhibit lower BMI and overt cachexia. Because the elderly may also present with “paucibacillary” disease—where bacterial loads are too low for traditional staining to detect—clinicians must rely heavily on high-sensitivity molecular tests to avoid diagnostic delay [60].

## 5. Endoscopy

**Figure 2:**
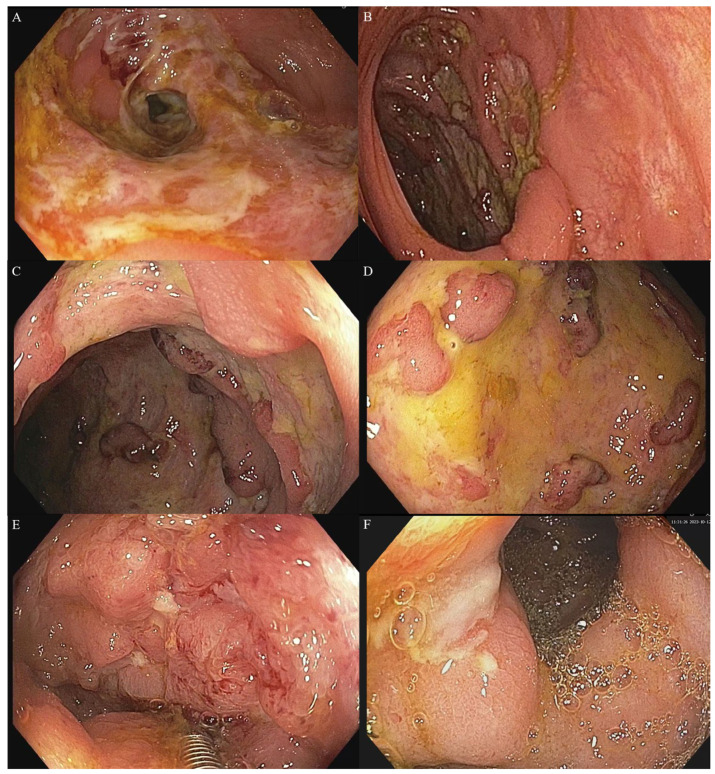
Endoscopic features in patients with ITB (**A**,**B**) and CD (**C**–**F**). (**A**) Circumferential stenosing ulcer (ITB); (**B**) Rat-like ulcer in a patient with ITB: (**C**) multiple, large ulcers with interposing inflamed mucosa (CD). (**D**) Geographic map aspect of colonic mucosa (CD). (**E**) Severe inflammation with cobblestone appearance and small ulcers in the caecum (CD). (**F**) Ulcer at the ileal valve in a patient with CD.

The main diagnostic technique for both CD and ITB is represented by ileo-colonoscopy. Although both diseases may be located in all gut segments, most cases involve the small and/or large bowel. Biopsy is essential for diagnosis, and ileo-colonoscopy allows multiple biopsies for both pathological and microbiological assessments [3]. The location, type, and aspect of the lesions may be suggestive of CD or ITB, although no lesion has been found to be strictly associated with CD or ITB; less than four segments involved, patulous ileocecal valve, circumferential (Figure 2A), rat-like (Figure 2B) or transverse ulcers were found more often in ITB [3,5,8,61], as opposite to aphthous, serpiginous or longitudinal ulcers, cobblestone aspect, skip lesions, and geographic map aspect (Figure 2C–F), which are more commonly observed in CD [3,5,8,9,61,62]. In children, deep or longitudinal ulcers, left-sided colon or multiple colonic segments, extraintestinal manifestations, and higher platelet count appear significantly more often in CD; isolated ileocecal involvement was more commonly observed in ITB [58]. In some studies, however, a significant proportion of patients with CD have only three segments involved [56]. Segmental ulcer and segmental colitis are the most commonly encountered ITB forms, while rectal involvement is rarely observed [3]. Jejunal and multiple-segment involvement are more often observed in CD [3]. The lesions in ITB tend to be continuous, variable in size, and bordered by irregular, “rat-bite-like” edges; ulcers are most often annular, and many patients show signs of intestinal narrowing. Less commonly, features such as scar diverticula or cold abscesses may appear in ITB, findings that immediately raise diagnostic suspicion [8].

Strictures may appear in both diseases with similar frequency but are shorter in ITB and longer in CD; a meta-analysis of 33 studies and 1969 patients with gastrointestinal tuberculosis has found a pooled prevalence of 27% [7]. Pulimood et al. found similar colonoscopic abnormalities in CD and ITB, with 60–70% of cases presenting ileocecal lesions [63]. Another study performed in China included 69 ITB and 107 CD patients and found that ITB patients more commonly had circular ulcers or rat-bite-like ulcers, persistent open ileocecal valve, tuberous and polypoid lesions, in contrast to fissure-shaped ulcers, cobblestone sign, more than four segments or rectum involvement, ileocecal valve stenosis, and mucosal bridges, which were more common in CD patients [64]. An endoscopic score was developed by Bae et al., with 65% sensitivity for CD and 97.5% for ITB, which increases to 96.3% by adding laboratory and radiological data [65]. Multiple biopsies are needed for both microbiological (bacteriology, PCR, and nucleic acid amplification test or NAAT) and pathological examination; the Indian Council of Medical Research recommends a minimum of six biopsies placed in sterile saline for bacteriology [3,8]. Table 1 details the main endoscopic findings used for differentiating ITB from CD.

Although the ileocecal region is the most affected area in both CD and ITB, gastroduodenal tuberculosis may rarely appear, and is expressed by epigastric pain, gastric outlet obstruction, hematemesis, or failure to thrive, often accompanied by systemic symptoms [6,52]. Endoscopic manifestations include ulcers, masses, nodularity, strictures, or extrinsic compression. Upper digestive endoscopy, videocapsule enteroscopy (VCE), and other device-assisted enteroscopy methods (push, balloon-assisted, or spiral enteroscopy) are useful for gastric or small bowel involvement [14,66]; deeper biopsy techniques and endoscopic ultrasound can improve diagnostic yield, particularly for submucosal or nodal involvement. Balloon-assisted and spiral enteroscopy are preferred because they may allow targeted biopsies; VCE is recommended in suspected CD without obstructive symptoms [67,68,69], has an incremental diagnostic value of 25–40% in CD [14], and may assess mucosal healing and early postoperative recurrence [14]. VCE cannot differentiate between CD and ITB because of the impossibility of performing biopsies; some case reports were published, and a study performed in South Korea on 19 CD and 15 ITB patients found that 10 or more ulcers, more than three segments involved, aphthous ulcers, and cobblestoning were more common in CD than in ITB [70]. Several case reports and case series were also available, and in a study of 106 patients performing balloon enteroscopy, 13 CD and 3 ITB were diagnosed [71].

Although some endoscopic findings are suggestive of either ITB or CD, an accurate distinction based solely on endoscopic criteria may be more difficult in a significant proportion of cases with ileo-colonic inflammation, as a mixture of endoscopic features may sometimes be encountered. In these cases, the association with clinical, cross-sectional imaging, pathological data, extraintestinal abnormalities, bacteriological findings, and interferon-gamma assays for TB may be helpful.

## 6. Cross-Sectional Imaging

**Figure 3:**
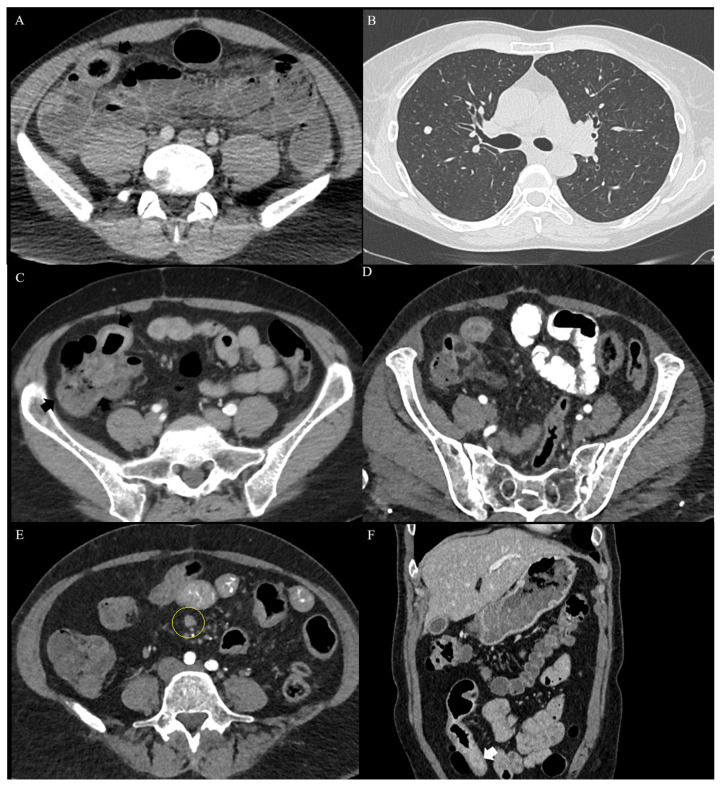
CT scan in patients with ITB (**A**,**B**) and CD (**C**–**F**). (**A**) Marked thickening of the ileocecal area (arrow), with dilated bowel loops (intestinal occlusion). Concomitant CT scan has shown pulmonary nodules with fibrosis (**B**); after bypass surgery, a favorable response to ATT confirmed ITB; (**C**) inflammatory mass (arrows) located in the ileocecal region (CD). (**D**) Same case: examination after oral contrast showed small bowel dilation. (**E**) Same patient: enlarged small abdominal lymph nodes (circle). (**F**) Coronal reconstruction (same patient) showed ileal stenosis (arrow) with pre-stenotic dilation (CD).

Cross-sectional imaging (CT or MRI, with or without enterography or enteroclysis) is a crucial method for diagnosing and differentiating between CD and ITB, because it allows evaluation of luminal abnormalities in both large and small bowels, which can be especially helpful in assessing small bowel involvement that is difficult to access with endoscopy [3,5]. Additionally, it enables assessment of extraluminal pathology, including abdominal lymph nodes, adjacent fatty tissue, and peritoneal inflammation [3,5,9,24,25]. CT/MRI enterography uses an oral contrast with follow-up sections, which allows small bowel distension and eliminates false wall thickening and false pathological enhancement; in contrast, enteroclysis uses a naso-jejunal tube for contrast administration, which can be less well-tolerated but more accurate for proximal small bowel lesions [24]. CT and MR enterography have similar sensitivity for the differentiation between ITB and CD; MR enterography is superior for strictures and fistula detection, and can better differentiate between fibrotic and inflammatory strictures, but may be limited by lower image quality because of motion artifacts and also by a higher price and limited availability in developing countries [24]. Imaging signs on CT or MRI enterography, similarly to those seen on colonoscopy (transverse or linear ulcers, short strictures, caecum retraction, patulous ileocecal valve, and narrowed terminal ileum), may be observed in patients with ITB (Figure 3A), and concomitant pulmonary TB may help in the diagnosis (Figure 3B). Conversely, features like aphthous, longitudinal, or deep ulcers, cobblestone appearance, skip lesions, asymmetrical wall thickening with predominant ileal involvement, mural stratification, involvement of multiple segments, and fistula presence (Figure 3C–F) are suggestive of CD [3,5,9,14,18,25,48,61,72,73]. Although typical for CD, cases of fistulizing ITB have been documented [69,74,75,76,77]. On MRI, circumferential but asymmetrical wall thickening, homogeneous mucosal enhancement, loss of stratification, short-segment stricture, shorter segments involved (fewer than four), necrotic, conglomerated or peripherally enhancing mesenteric lymph nodes, ascites, and omental and peritoneal disease are more commonly observed in ITB [78]. In two studies examining CT enteroclysis or CT enterography, homogeneous bowel wall thickening with confluent involvement and necrotic or calcified lymph nodes were more commonly associated with ITB. Meanwhile, stratified wall thickening with an intervening fat layer, mucosal enhancement, skip lesions, long segment or left colon involvement, fibrofatty proliferation, and the “comb sign” were more commonly associated with CD [24,25,79]; however, neither of the sets of signs are strictly pathognomonic for either condition. In patients with small bowel perforation, bowel wall thickening above 10 mm, omental thickening, and intra-abdominal lymph nodes larger than 10 mm were more common in ITB cases, in contrast with mesenteric fat infiltration, which was more typical in CD [80].

Extraintestinal signs can also orient for CD or ITB; pulmonary TB, ascites, and necrotic lymph nodes suggest ITB, and the comb sign (prominent mesenteric vasculature adjacent to a dilated loop) or jejunization of the ileal vasculature, mesenteric fatty proliferation, and stranding are more often encountered in CD [3,18,44,81,82]. Pulmonary TB is present in up to 25% of patients with ITB; thoracic CT may increase the accuracy of ITB diagnosis from 26.2% to 56.9% [83], although cases of CD associated with active pulmonary TB or concomitant ITB-CD infections have also been noted [84,85]. A meta-analysis of six studies, comprising 417 CD and 195 ITB patients, found that the most accurate features in ITB were necrotic lymph nodes, and the comb sign and skip lesions for CD [86].

Some recent studies have examined the role of ultrasound in diagnosing inflammatory bowel disease [3,72]. A thickened bowel wall above 3 mm, an enhanced Doppler flow associated with inflammation presence, and mesenteric fat hyperechogenicity were described in cases of CD [3,72]; in contrast, ascites and regional enlarged lymph nodes were more often found in patients with ITB [3,5,8,14,24]. Only a few studies assessed the role of endoscopic ultrasound (EUS) in the differentiation between CD and ITB; in a study, thickened submucosa with a slightly high echo level and a visible layer was noted in CD, and thickened mucosa with a high or slightly high echo level and a visible layer was noted in ITB. Accuracy rates of between 83.6 and 89.3% were noted for both diseases [87]. The accessibility of lesions for EUS examination (both diseases are commonly located in the ileocecal region) may limit the applicability of EUS for differentiation.

Newer techniques, such as contrast-enhanced ultrasound (CEUS), dynamic contrast-enhanced MRI (DCE-MRI), apparent diffusion coefficient (ADC), and hybrid PCT/MRI, may add supplementary and more accurate data for differentiation; however, more dedicated studies are needed [3]. Because the microvascular environment differs between CD and ITB, newer techniques such as perfusion CT may also help to discriminate between the two diseases. A study evaluating blood flow, permeability, and mean transit time (MTT) in 11 ITB and 15 CD patients has found significant differences between CD and ITB, with 100% sensitivity and specificity for blood flow and permeability, and 61.5–100% sensitivity and 70–100% specificity for MTT [88].

Cross-sectional imaging methods can help evaluate treatment response in both diseases and, therefore, assess a lack of or insufficient response as a possible indication of misdiagnosis between CD and ITB [3,89]. Intestinal ultrasound, ileo-colonoscopy, and CT/MRI can assess mucosal healing with comparable accuracy in a meta-analysis of 39 studies including CD [3]; a reduction in intestinal thickness by more than 25% or more than 1–2 mm associated with a reduction in Doppler signal is considered suggestive of CD. The accuracy is similar for ITB; 100% sensitivity and 50% specificity were noted in a study of 20 patients [3]. A combination of CT enterography and gastrointestinal ultrasound significantly increases the accuracy of response evaluation [89].

The higher ratio of visceral fat to either total fat (VF/TF) or subcutaneous fat (VF/SF) or a combination of increased VF and a long segment involved (>4 cm) was confirmed in several studies as being predictive of CD [90,91,92,93]; in children, the predictive value of VF/SF ratio was observed particularly in males [93]. Table 2 summarizes the main cross-sectional features used for differentiation.

## 7. Pathology

**Figure 4:**
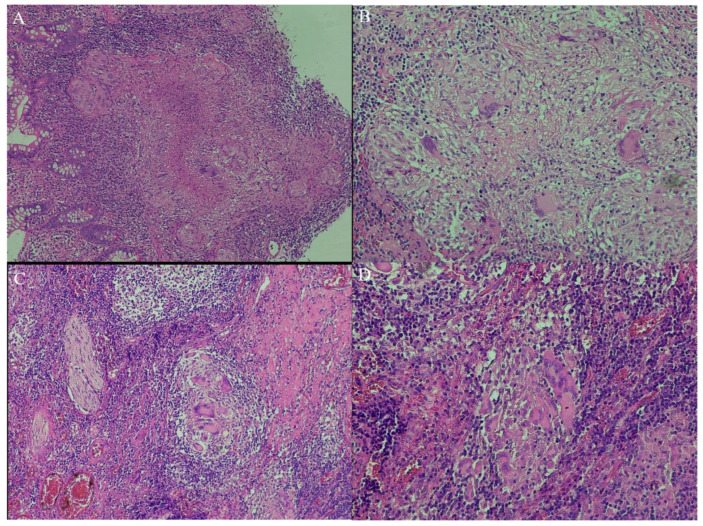
(**A**) ITB, HE stain, 10× magnification (left) and 20× (right). (**B**) ITB: well-organized, confluent epithelioid granuloma with central caseous necrosis, a large number of Langhans-type giant cells. (**C**,**D**) Crohn’s disease, HE stain, 10× magnification (**C**) and 20× (**D**). Small, poorly demarcated, non-caseating epithelioid granuloma consisting of aggregates of epithelioid histiocytes with multinucleated giant cells.

The role of pathology in distinguishing between CD and ITB is important; however, many features are common to both diseases. Traditional pathological findings of ITB and CD include ulcers, focal crypt abnormalities (distortion, shortening, or branching, with lower density and irregular surface) adjacent to normal crypts, discontinuous transmural inflammation, and granulomas [5,94]. Differences regarding the presence of various pathological features were also noted between colonoscopic and surgical specimens, as colonoscopic biopsies are more superficial and usually contain only the mucosal layer [3,94,95]. Granuloma presence is considered pathognomonic for both diseases; 10–80% of ITB cases and 15–65% of CD cases had granuloma in biopsy specimens, with higher rates in surgical than endoscopic specimens [3]. Multiple, larger and confluent caseating granulomas that can be located in all layers and in lymphoid tissue, associated with macrophage-lined ulcers and excessive disproportional submucosal inflammation (Figure 4A,B), are suggestive of ITB [2,3,5,8,14,18,61,62,96,97]; caseating granulomas were, however, uncommon in CD [98]. A study of 41 granulomas from 4430 perianal fistulae found that granulomas in patients with CD were predominantly without foreign body giant cells, in contrast to non-CD granulomas, which contain foreign body giant cells in 87.5% [99]. In CD, deep ulcers with mucosal architecture distortion, cryptitis with abscesses, and moderate or severe inflammation are often encountered; granulomas are usually small (Figure 4C,D) [3,8] and located in both the intestinal wall and in 10% of regional lymph nodes (almost always only in association with intestinal inflammation [3,14,100]. Microgranulomas, defined as a loosely organized collection of epithelioid histiocytes without other defining elements for granulomas, were observed in the mucosal layer in 40% of CD patients [63]. In a meta-analysis [101], caseating confluent granulomas and ulcers delineated by epithelioid histiocytes were the most predictive pathological abnormalities for differentiation between CD and ITB, and in another meta-analysis by Limsrivilai et al., confluent, large, submucosa, and multiple granulomas per section, together with surrounding lymphocyte cuffing, were the most suggestive abnormalities for ITB [54]. In a meta-analysis published by Du et al., confluent granulomas and ulcers lined by epithelioid histiocytes rendered high accuracy for ITB diagnosis [101].

A combination of pathological signs may increase predictive value; however, in two studies, pathological features associated with high suspicion for ITB are present in only half of the cases [4,48]. In a pan-Indian survey of pathologists focused on IBD diagnosis, 86.6% noted that large confluent epithelioid granulomas in deep layers favor ITB diagnosis, but only 8.6% assessed granulomas larger than 400 µm as characteristic for ITB [102]. A higher number of biopsies (8) may increase the accuracy of ITB diagnosis [22,97,103]. Table 3 details the main pathological findings useful for discriminating between CD and ITB.

Some studies reported the role of immunohistochemistry in the differentiation, with CD73 being more specific for TB granulomas [105,106]. In a study, the macrophage M1 polarization was more common in CD, especially in the presence of granulomas, whereas M2 polarization was more common in ITB granulomas [107]. In another approach, trichromic Masson staining, together with second-harmonic generation and two-photon excited fluorescence, demonstrated that collagen fibers and fiber deposits are more common in ITB compared to CD [108]. A study using immunohistochemistry with anti-VP-M660 antibody targeting the 38 kDa antigen of *Mycobacterium tuberculosis* noted positive immunostaining in 33/45 ITB cases but only 2/28 CD patients (73% sensitivity, 93% specificity) [109]. A study evaluating syndecan-1 and heparanase abnormalities in serum and colonic biopsies in patients with both ITB and CD has shown abnormalities limited to the CD group only; syndecan-1 was significantly decreased in mucosa and increased in serum in CD patients, with heparanase level elevated in both tissue and serum samples [110].

## 8. Gamma-IFN Assays and Serology

Positive tuberculin skin test (Mantoux test) and interferon-gamma release assays (IGRAs) are important arguments for ITB diagnosis, although they have limitations in countries with high TB infection prevalence. The Mantoux Test is positive in past or latent TB infection; high prevalence of previous occult infections in high-endemic areas makes the test less sensitive for differentiation [3,8], although past infection makes the diagnosis of ITB more probable. Previous TB vaccination and anergic immunocompromised status may further complicate the interpretation of the Mantoux test [8], and positive results in only 50% of patients with ITB underline the low predictive value [8]. Even in Western countries, latent TB infection can be significant; in Spain, an estimated 12.5–33.5% of the population has latent infection [8]. A negative test does not completely rule out ITB, as many studies have found [3]. IGRA evaluates the release of interferon-gamma after stimulation with two synthetic peptides (early secretory antigenic target-6 or ESAT-6, and culture filtrate protein-10 or CFP-10) which are similar to proteins present in *Mycobacterium tuberculosis* and absent in BCG vaccine and most other *Mycobacterium* species (except for *M. Kansai*, *marinum and szulgai*), IGRA is more accurate than the Mantoux test, with both sensitivity and specificity higher than 80%, and fewer false-negative results in immunocompromised patients [3,8,14,111]. Several IGRA tests (such as QuantiFERON TB Gold—Cellestis Ltd., Melbourne, Australia, and T-SPOT.TB—Oxford Immunotec Global PLC, Abingdon, United Kingdom) are currently available; both have negative predictive values above 90%, which makes them ideal for excluding ITB [8,61,112]. The level of TB-IGRA can help to differentiate, with values of 100 pg/mL or higher associatied with 88% sensitivity and 74% specificity for ITB diagnosis; clinical judgment may be important for values between 14 and 99 pg/mL [113]. However, poor accuracy, coupled with both false-negative and false-positive results, was recorded in high-endemic areas such as India [114]. A study of 39 cases of small bowel perforation in both ITB (18 cases) and CD (22 cases) found that only 27.8% of patients with ITB had a positive QuantiFERON-TB Gold test [80]. Some meta-analyses are available today; a meta-analysis published in 2013 included five studies, two from China and three from South Korea, and found 74% sensitivity, 87% specificity, and a 0.92 AUC [115], while another meta-analysis including eight IGRA and four ASCA (anti-Saccharomyces cerevisiae antibodies) studies has found pooled sensitivity, specificity, positive likelihood ratio, and negative likelihood ratio of 81%, 85%, 6.02, 0.91, respectively, and AUC of IGRA for ITB of 0.92, and 33% sensitivity, 83% specificity, and 0.58 AUC for ASCA in CD [116]. Another meta-analysis of 12 studies from Asia showed a pooled sensitivity of 82.8% and a pooled specificity of 86.7% for IGRAs in differentiating between ITB and CD, with a positive likelihood ratio of 6.870, a negative likelihood ratio of 0.171, and an AUC of 0.939 [117]. T-SPOT.TB showed higher sensitivity than QuantiFERON-TB Gold for ITB diagnosis in two studies [116,118].

The resurgence of tuberculosis in many regions is inextricably linked to the HIV epidemic. In HIV-positive individuals, ITB often presents as part of a disseminated infection rather than an isolated gastrointestinal disease [57]. These patients are commonly “anergic,” meaning they may produce false-negative results on traditional Tuberculin Skin Tests (Mantoux). However, IGRAs such as QuantiFERON-TB Gold remain more reliable in this sub-population, showing fewer false negatives even when the immune system is compromised [119,120].

Specific antibodies, such as ASCA, anti-glycan antibodies, anti-outer-membrane porin C (OmpC), anti-flagellin, anti-zymogen granule GP2, or anti-I2 (Pseudomonas fluorescens-associated senescens), were proposed in studies for the differentiation, but currently lack sufficient sensitivity and specificity for clinical significance [3,5,8,51,121,122]; higher levels and higher prevalence of ASCA IgG antibodies were noted in CD compared to ITB patients in some studies [42,123,124]. The combination of a positive ASCA test with a negative QuantiFERON TB Gold can significantly increase CD diagnosis sensitivity and specificity [8]. A study published in 1995 evaluated the role of antibodies against cord factor using enzyme-linked immunosorbent assay (ELISA) for ITB, with 23 of 27 ITB patients being positive compared with none of the 16 CD patients [125]. 

## 9. Microbiology and Nuclear Testing

The detection of *Mycobacterium tuberculosis* by bacteriology or nuclear testing represents the gold standard in ITB; however, the paucibacillary nature of extrapulmonary tuberculosis may complicate diagnosis [1,5,51]. Bacteriological tests, such as acid-fast bacilli (AFB) tests, are limited by low sensitivity (<5%) [1,3,14]. Traditional culture-based diagnostics (Lowenstein–Jensen medium, and other agar-based or liquid medium) require time but allow testing for drug susceptibility [3,14,18]; much faster automated techniques, such as BACTEC, Mycobacteria Growth Indicator Tube (MGIT), MB/BacT mycobacterial detection system, or ESP culture system II, have demonstrated higher sensitivity and quicker results than Lowenstein–Jensen culture [2,3,5,14]; BACTEC sensitivity for ITB is reported to be lower in some studies [14,126], but high specificity and positive predictive value are usually noted, with an accuracy of 48.6% [126]. An increased number of biopsies can significantly improve diagnostic yield [2]. Fecal mycobacterial culture was a reliable test in some reports [127].

PCR-based tests for *Mycobacterium tuberculosis* rely on the amplification of oligonucleotides highly specific to the *Mycobacterium tuberculosis* genome (IS6110, MBP64, or Protein B); they are quicker and more sensitive than traditional bacteriological methods [3,14,128]; although taken alone, they are not diagnostic for ITB. Currently, DNA targeting, RNA targeting, and gene amplification assays are available for PCR detection of ITB [2]; the low sensitivity because of the small amount of tissue provided by biopsies represents a major limitation, but a major advantage is related to the small amount of bacterial DNA needed for the detection (six copies/mL) [129]. Several techniques are available today, such as loop-mediated isothermal amplification assay, PCR, multiplex PCR IS6110, MBP64, Protein B, nested PCR, real-time PCR, True-NAT MTB plus, and GeneXpert^®^ MTB/RIF or Xpert Ultra [130,131,132,133]; the specificity was close to 100%, but sensitivity ranges from 23 to 70%, and a systematic review and meta-analysis have found a pooled sensitivity of 47% [134]. Loop-mediated Isothermal Amplification Test for TB (TB-LAMP) is based on amplification of *Mycobacterium tuberculosis* DNA, targeting gyrB and IS6110 genes [2]; the detection rate is higher than multiplex PCR [2]. In two studies evaluating performance of Xpert-Mtb/Rif in the diagnosis of ITB in patients with ileo-colonic ulcers [130] and in diagnosis of ITB, the sensitivity, specificity, PPV, and NPV were 32%, 100%, 46.88%, 100%, and 57.5% [130], and 8.1%, 100%, 100%, and 64.2% [132], respectively, with a low sensitivity in both studies. A combination of Xpert-Mtb/Rif with MPT64 tuberculosis protein increased the sensitivity from 33.3% to 50% [131], whereas a combination of Xpert-Mtb/Rif with pathology was associated with a 97% diagnostic value [135]. Another PCR technique, fluorescent quantitative PCR, was tested in fecal samples and tissue biopsies, with 82.8% positivity in fecal samples and 55.2% of biopsies, compared to 8.3% and 5.6% in CD patients [136]. The combination of fecal and biopsy PCR had a diagnostic yield of 97.5% [137]. A systematic review and meta-analysis published in 2017 analyzed RT-PCR performance in 25 studies including patients with extrapulmonary tuberculosis, and found a pooled sensitivity of 70% (with three studies showing values between 20 and 40%), 99% specificity, and a 0.96 AUC for diagnosis [129]; however, most of the included studies analyzed other extrapulmonary locations outside the bowel, which raises concerns about the extrapolation of the estimated PCR performance to ITB patients. Another 2017 meta-analysis included nine studies and found a pooled sensitivity of 47%, a pooled specificity of 95%, and an AUC of 0.9311 [134]. A systematic review and meta-analysis assessing the performance of Xpert MTB/TIF for abdominal and ITB has shown low sensitivity for ITB, with a pooled sensitivity and specificity of 23% and 100%, and an AUC of 0.499 [138]. The True-NAT MTB plus appears superior to both Xpert and multiplex PCR, with 70% sensitivity and 100% specificity in a study of 30 ITB and 20 controls [139].

A more recent and promising molecular test is targeted next-generation sequencing; a few studies have found high sensitivity and specificity rates (74–88.2% and 94.1–100%) [140,141,142]. A recently published approach, using nano-based assay, a SYBR Green magnetic bead-coupled gold nanoparticle-based real-time immuno-polymerase chain reaction (MB-AuNP-RT-I-PCR) for the quantitative detection of *Mycobacterium tuberculosis* MPT-64+CFP-10 proteins in 51 extrapulmonary tuberculosis patients and 49 controls, showed 88.2% sensitivity and 100% specificity [143].

The bacteriological confirmation of *Mycobacterium tuberculosis*, although rarely encountered, permits a definite ITB diagnosis; in most cases, however, only a positive IGRA test argues for ITB. The low sensitivity and moderate negative predictive value may impact the accuracy of both IGRA and nuclear testing.

## 10. Therapeutic Response

The antitubercular treatment (ATT) with response evaluation may be used for the differentiation between CD and ITB; the toxic effects of ATT (especially liver) and risk of drug resistance [18,20,144], and also the risks of delayed therapy and both penetrating evolution and stricture formation (with subsequent risk for surgery) may impact this type of strategy for differentiation [3,18,144,145,146,147]. Some cases of CD may also express improvements in symptoms and inflammatory markers during ATT, which can delay an accurate diagnosis [3,84,148]; a latent TB-associated infection or an independent anti-inflammatory effect of isoniazid may explain this finding [149]. Immunosuppressive therapy, corticotherapy, and biological treatment (except for anti-integrins) may increase the risk of reactivation or disseminated TB [3,8,150,151,152,153,154,155,156,157], perforation [8,158,159], need for surgery [21,160,161,162,163,164,165], and even increase mortality [3,20,21,166], and are not recommended in patients from TB-endemic regions without a definitive TB exclusion [3]. Even patients who initially test negative for TB may develop active disease during the course of anti-TNF therapy. Newer biologics such as vedolizumab (an integrin antagonist) are reported to have a lower risk of TB reactivation than anti-TNF agents, making them a safer alternative in TB-endemic regions. In cases where a patient develops active TB during treatment, switching to a drug with a different mechanism, such as an interleukin-17 inhibitor, may be necessary after completion of antimycobacterial therapy.

Conflicting data on the accuracy of clinical assessment of therapeutic response have suggested the need for clearer markers; ulcer healing is the gold standard, but non-invasive markers (such as PCR or calprotectin monitoring or imaging markers for response) were also proposed [167,168,169]. Some guidelines (such as the Asia Pacific guideline and the World Gastroenterology Organization Global guideline) proposed an empiric ATT of 2–3 months in complex and uncertain cases, with clinical response (symptom resolution and weight gain) and control colonoscopy used for assessment [3,12,170]. We must have in mind that the empirical ATT can delay proper therapy in patients with CD and may, therefore, increase the risk of complications [144,145,146]. A profibrotic effect of ATT has also been noted [1,145,146]. A significant proportion (37%) of CD patients have shown clinical improvement during ATT, although endoscopic healing was much rarer [8]. Studies that included a follow-up period of up to one year have shown a poorly sustained response to ATT in patients with CD [5]. Multi-drug resistant ITB may have a similar evolution to CD during ATT [5,14,169], although the prevalence is reported to be low (between 5 and 13%) [5,14]. Recurrent ITB cases have been noted, and it is sometimes difficult to determine whether they represent real recurrences of ITB or CD misdiagnosed as ITB [14]. In a study of 89 patients with definite CD (26 cases), definite ITB (21 cases), and indeterminate diagnosis (42 cases), after 8 weeks of ATT, another 28 ITB cases (which respond to ATT) and another 14 CD cases (not responding to ATT) were diagnosed [171]; symptomatic improvement during ATT was associated with ulcer healing, but 23.6–40% of strictures persist in patients with ITB despite 6 months of ATT, which can make it harder to differentiate from CD [171,172].

## 11. Computerized Models and Artificial Intelligence

Many computerized models, including demographic, clinical, colonoscopic, CTE, pathological, and serological data [5,27,30,49,54,65,173,174,175,176,177,178,179,180,181,182,183,184,185,186,187,188,189,190], were proposed and studied in the available literature (Table 4); however, a universal application is still debatable without multicenter testing. Studies including clinical, endoscopic, and pathological data have demonstrated good sensitivity and specificity, with bloody stools, weight loss, sigmoid or rectal involvement, pulmonary tuberculosis, ascites, macroscopic appearance, and the Mantoux test being the most commonly reported parameters. Some combined models were proposed by Zhang (perianal disease, transverse and rodent-like ulcer, skip lesions, comb sign, and patulous ileocecal valve), Kedia (CT scan with long segment affected, ileocecal area involvement, and enlarged lymph nodes more than 1 cm), Zeng (T-spot positive test, cobblestone aspect, comb sign, and granuloma), Lee YJ (type of ulcers, less or more than four segments involved, patulous ileocecal valve, pseudopolyps, cobblestoning, and ano-rectal lesions), Lee Y (abdominal pain; perianal abscess; ileus; hepatobiliary disease; tuberculosis history; CRP; T-SPOT; segmental lesions; longitudinal ulcer; involvement of jejunum, ascending colon, rectum, and perianal fistula), Jung (age, gender, diarrhea, ring-shaped ulcer, longitudinal ulcer, sigmoid involvement, and suspicious radiological pulmonary tuberculosis), and Makharia (weight loss, bloody stools, sigmoid involvement, and enhanced colitis at pathology), with good performance [30,174,177,179,180,182,183]. Li et al. [185] has shown that the clinical model (hematochezia, intestinal surgery, perianal diseases, pulmonary tuberculosis, ascites, and positive PPD skin test) had 90.3 sensitivity, 76.8% specificity, 83.3% accuracy, 80.7% positive predictive value, and 88% negative predictive value, while the endoscopic-based model (rectum involved lesions, longitudinal ulcer, cobblestone appearance, fixed-open ileocecal valve, transverse ulcer, and rodent ulcer) had 82.9% sensitivity, 82% specificity, 82.5% accuracy, 82.9% positive predictive value, and 82% negative predictive value. Most studies, however, are limited by small sample size, lack of a validation group, and non-linear relationship between variables or complex formulas used in nomograms.

Several artificial intelligence-based models have also been tested, with good performance; the lack of external validation is a limitation of this approach. A machine-learning-based algorithm using the XGBoost method, which included nine parameters (abdominal signs, bloody stools, intestinal surgery, intestinal dilation, comb sign, knot, PPD, ESAT-6, and CFP-10), noted an AUC of 0.891, with 81.3% sensitivity and 96.9% specificity [176]. A deep learning method, based on endoscopic images of 211 CD, 299 intestinal Behcet disease, and 217 ITB patients, has proved a moderate accuracy of the algorithm for discriminating the three diseases (all images: 65.15% vs. typical images: 72.01%, *p* = 0.024), with convolution neural network (CNN) being useful in the differentiation between colonoscopic images (AUC ranging from 0.7846 to 0.8586) [186]. A 2024 systematic review of nine studies that used retrospective databases (five studies included endoscopy-based AI, one included radiology-based AI, and three included multiparameter-based AI) showed fair accuracy, ranging from 69.6 to 100% [191].

A limited number of multicenter studies or meta-analyses are available today. A meta-analytic Bayesian model (Supplementary File S2: Bayesian meta-analysis principles) including age, clinical data, endoscopy, and laboratory findings was proposed by Limsrivilai and had both sensitivity and specificity above 90% [54], with a validation cohort confirming the findings. A multicenter validation study (performed in five hospitals from Thailand and Hong Kong) has included 199 patients with either CD or ITB and fully available parameter recordings, and analyzed the performance of Lee, Makharia, Jung, and Limsrivilai models; AUC values were 0.713, 0.637, 0.862, and 0.853 for combined clinical–endoscopical scores, with the last two scores being similar statistically (*p* = 0.52) and by adding pathological parameters, the AUC of Limsrivilai model increases to 0.887, significantly higher than that for the Jung and Makharia model [192]. Until a model with enough accuracy, multicenter worldwide validation, and acceptance emerges, the described models will only have limited use for differentiation.

**Figure 5:**
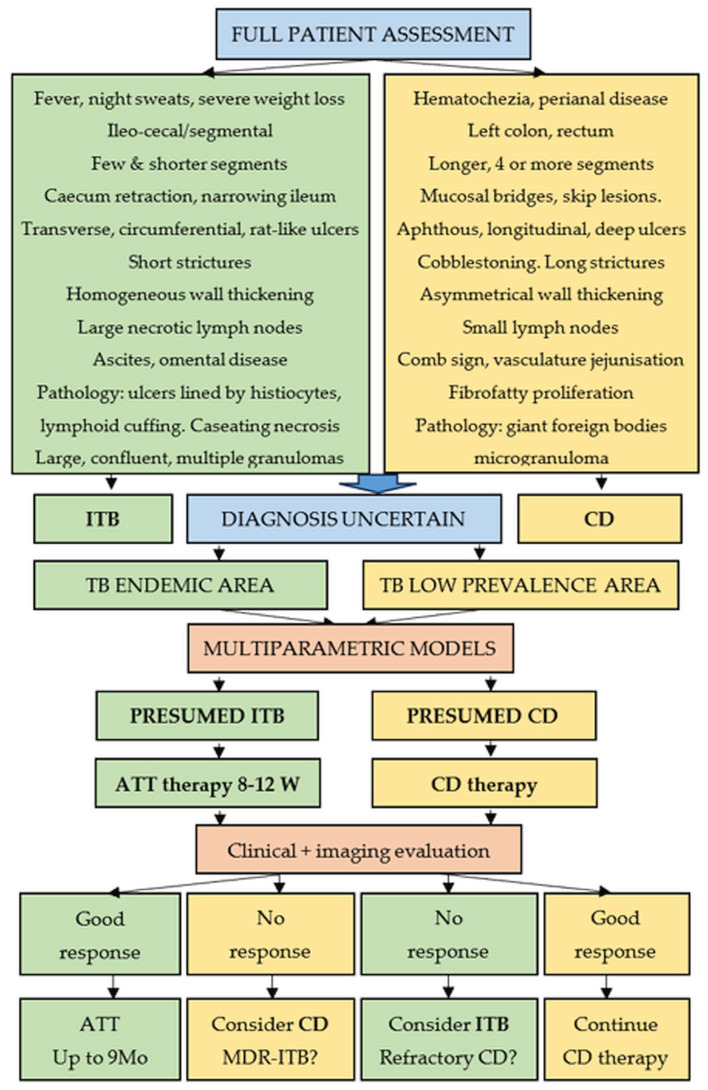
Management strategy for uncertain cases of CD or ITB.

An algorithm for managing uncertain cases of ITB or CD is presented in Figure 5.

**Table 4:** Models for the differentiation between CD and ITB.

Author, Year	Country	Patients	Parameters	Model	Performance	Validation Set
Watermeyer, 2018 [27]	South Africa	68 ITB-48 CD	HIV positivity, isolated colitis, and absence of extraintestinal manifestations	Multivariate analysis	NO DATA	NO
Zhang, 2015 [30]	China	31 ITB-92 CD	Clinical (diarrhea, night sweat, perianal disease), colonoscopic (transverse, longitudinal, or rodent-like ulcers and patulous ileocecal valve), CT enterography (skip lesions, asymmetric involvement, ileocecal valve), T-SPOT	Multivariate analysis $p=\frac{1}{1+e^{-(-1.279+4.814x_{13}-5.151x_{25}-3.622x_{27}+5.399x_{34}-3.897x_{37}+4.477x_{42})}}$	Sn 97.8% Sp 96.8% ACC 97.6% PPV 98.9% NPV 93.7%	NO
Huang, 2015 [49]	China	40 ITB-25 CD	12 features (longitudinal ulcers, ring ulcers, ulcer scars, nodular hyperplasia, cobblestone-like mucosa, intestinal diseases, intestinal fistula, target sign, the comb sign, night sweats, tuberculin skin test, T-SPOT.TB).	Simple score (features supporting CD +1, supporting ITB scored −1)	AUC 0.997	NO
Limsrivilai, 2017 [54]	Multicentric	38 studies 2117 CD-1589 ITB	CD: male, hematochezia, perianal disease, intestinal obstruction, extraintestinal manifestations; longitudinal ulcers, cobblestone, luminal stricture, mucosal bridge, rectal involvement; focally enhanced colitis; CT enterography (asymmetrical wall thickening, wall stratification, comb sign, fibrofatty proliferation ITB: fever, night sweats, lung involvement, ascites; transverse ulcers, patulous ileocecal valve, cecal involvement; confluent or submucosal granulomas, lymphocyte cuffing, ulcers lined by histiocytes; CTE short segmental	Bayesian meta-analysis	Sn 90.9% Sp 92.6% Acc 91.8%	Not appliable
Bae, 2017 [65]	South Korea	40 ITB-40 CD	Colonoscopic, laboratory, and radiologic factors	Multivariate analysis	Combined score increased accuracy from 81.2% to 96.3%. AUC 0.990–0.981	YES
Cheng 2024 [173]	China	85 ITB-245CD	Radiomics features	Deep learning CNN followed by LASSO regression	The arterial-venous combined deep learning radiomics model had AUCs of 0.885, 0.877, and 0.800	YES (2 datasets)
Li 2022, [174]	China	105 ITB-227 CD	13 parameters in the LASSO model (abdominal pain; perianal abscess; ileus; hepatobiliary disease; tuberculosis history; CRP; T-SPOT; segmental lesions; longitudinal ulcer; jejunum involvement; ascending colon involvement; rectum involvement; perianal fistula)	LASSO regression vs. classical regression	Sn 84.4 vs. 65.9% Sp 80.6 vs. 89.9% PPV 82.3 vs. 88.5% NPV 82.9 vs. 68.9% AUC 0.887 vs. 0.811,	YES
Shu, 2024 [175]	China	123 ITB-118 CD	51 demographic, clinical, laboratory, colonoscopy, and pathology parameters. Best performance: T-spot, pulmonary tuberculosis, and age at onset	Six machine-learning methods tested (XGBoost best)	Sn 87.1% Sp 83.3% Acc 86% AUC 0.946	NO
Weng, 2022 [176]	China	40 ITB-160 CD	Nine variables (intestinal surgery, abdominal, bloody stool, PPD, knot, ESAT-6, CFP-10, intestinal dilatation, and comb sign)	10 Machine-learning methods (XBoost best)	Sn 75.2–81.3% Sp 95.3–96.9% AUC 0.853–0.891	YES
Zeng 2022 [177]	China	43 ITB-90 CD	T-SPOT positive, cobblestone appearance, comb sign, and granuloma	Multivariate analysis LogitP = 0.340 − 5.457 × T-SPOT positive + 3.353 × cobblestone appearance + 4.436 × comb sign − 2.967 × granuloma	Sn 94.4% Sp 93% Acc 94% AUC 0.988	NO
Zhu, 2021 [178]	China	93 CD-67 ITB	2 clinical and 9 radiomics features for the region of interest (ROI) of the ileocecal region (CT enterography), filtered by the gradient boosting decision tree (GBDT). The radiomics score was calculated using the radiomics signature-based formula	Multivariate analysis	AUC 0.96 (95% CI: 0.93–0.99) in the training cohort, 0.93 (95%CI: 0.86–1.00) in validation cohort.	YES
Lee, 2006 [179]	South Korea	44 ITB-44 CD	Four parameters were more common in CD (anorectal lesions, longitudinal or aphthous ulcers, and cobblestone appearance), and four other parameters (involvement of fewer than four segments, a patulous ileocecal valve, transverse ulcers, and scars or pseudopolyps) were more common in ITB	Decision Tree Analysis	Correct diagnosis 87.5%	NO
Kedia [180]	India	50 ITB-54 CD	Colonoscopic and CTE (ileocecal involvement, long-segment involvement, lymph node ≥1 cm)	Simple score (1 p each)	0–1p: Sp 100–87%, 100–76% PPV (ITB). 2–3p: Sp 68–90%, PPV 63–80% (CD)	YES
Jung 2016 [182]	South Korea	99 ITB-162 CD	Age, gender, diarrhea, ring-shaped or longitudinal ulcer, sigmoid involvement, suspicious radiological pulmonary TB	Multivariate logistic regression	Sn 98%, Sp 92.4% PPV 88.9% NPV 98.6%	YES
Makharia 2009 [183]	India	53 ITB-53 CD	Blood in stool, weight loss, sigmoid involvement, and focally enhanced colitis	Multivariate analysis	Sn 83–90% Sp 79.2–60% Acc 81.1% AUC 0.9089–0.892	YES
Hilmi, 2022 [184]	Malaysia	30 ITB-134 CD	Close contact TB, AFB/PCR/culture positive, multiple/large/caseating granulomas, ascites, lymph nodes, pulmonary findings, TST/IGRA positive + ATT	Decision tree algorithm	Sn 96.7% Sp 88.1% Acc 89.6% NPV 99.1% PPV 64.4%	NO
Kim, 2021 [186]	South Korea	217 ITB-211 DC	Colonoscopy images	Convolution Neural Network Analysis	AUC 0.7846–0.8586 For typical images, AUC ranged from 0.8211 to 0.9360	NO
Gong, 2023 [187]	China	105 ITB-CD	Clinical–radiomic (one radiomic signature from the intestinal wall, one radiomic signature from the LN, involved bowel segments on CTE, and a longitudinal ulcer on endoscopy)	Multivariate analysis	AUC 0.975 in the training cohort and 0.958 in the validation cohort.	YES
Zhao, 2014 [188]	China	47 ITB-141 CD	Clinical (hematochezia, perianal disease, ascites, night sweats, pulmonary TB), CTE (left colon, asymmetric involvement, abscess, comb sign, lymph nodes along the right colic artery, contracture of the ileocecal valve, fixed patulous ileocecal valve, and lymph nodes with central necrosis), and TST	Multivariate analysis	Clinical/CTE features Sn 94.3–96.5% Sp 80.4–93.6% Acc 91.0–95.7% PPV 93.7–97.8% NPV 82.6–89.8%	NO
He, 2019 [189]	China, Israel	69 ITB-143 CD	Age, transverse ulcer, rectum involvement, skipped involvement of the small bowel, target sign, comb sign, and IRGA	Multivariate analysis	Sn 86.8% Sp 90.9% PPV 97.1% NPV 66.7% Acc 87.8%	NO
Yu, 2012 [190]	China	43 ITB-53 CD	Night sweats, longitudinal ulcers, and granulomas	Multivariate analysis	AUC 0.8642	NO
Lu 2021 [193]	China	106 ITB-106 CD	IGRA, ≥4 segments involved, longitudinal, circular, or aphthous ulcer	CART	Sn 90.91% Sp 86.36% NPV 90.48% PPV 86.96% Acc 88.64%	YES
Wu 2018 [194]	China	86 ITB-153 CD	Perianal disease, pulmonary involvement, longitudinal ulcer, left colon, and ratio of tuberculosis-specific antigen to phytohemagglutinin	Multivariate analysis	AUC, Sn, Sp, and accuracy were 0.975–0.950, 96.7–88.5%, 90.7–93.5%, and 92.8–91.7% in the training and validation sets.	YES

Sn = sensitivity, Sp = specificity, PPV = positive predictive value, NPV = negative predictive value, Acc = accuracy, ITB = intestinal tuberculosis, CD = Crohn’s disease, LN = lymph nodes, IGRA = interferon-gamma release assay, ATT = anti-tubercular therapy.

## 12. Conclusions

The differentiation between Crohn’s disease and intestinal tuberculosis remains a formidable clinical challenge, especially in developing countries where the epidemiological transition has led to a significant overlap between these two conditions. Clinical particularities are less often suggestive for differentiation; fever, night sweats, and severe weight loss are more suggestive for ITB, and a more indolent evolution and perianal disease suggest CD. Endoscopic, cross-sectional imaging, and pathology features may overlap in roughly 60–70% of cases, with granuloma characteristics (if present) being the most useful criterion for differentiation. The paucibacillary nature of ITB often limits the sensitivity of traditional bacteriological and molecular methods, but the confirmation of Mycobacterium tuberculosis makes the diagnosis certain. Tuberculin tests and IGRA may signify either previous or current infection and cannot be used as exclusion criteria. The emergence of computerized models and artificial intelligence—including XGBoost and deep learning algorithms—offers a promising avenue for more accurate, automated differentiation. A multidisciplinary approach integrating clinical, endoscopic, and radiomics data is essential to avoid misdiagnosis, prevent unnecessary antitubercular therapy, and ensure timely management of CD complications.

## Figures and Tables

**Table 1:** Main signs for the differentiation between ITB and CD at ileo-colonoscopy.

Findings	ITB	CD
Site	Segmental colitis Isolated ileocecal involvement	Left colon Rectal (rarely)
**Segments involved**	Fewer	4 or more
**Ulcers**	Transverse, circumferential Rat-like	Longitudinal, serpiginous Aphthous, segmental
**Skip lesions**	Absent	Present
**Cobblestoning**	Absent	Present
Strictures	Short	Long
Particular aspects	Scar diverticula Patulous ileocecal valve Narrowed terminal ileum	Mucosal bridges

ITB = intestinal tuberculosis, CD = Crohn’s disease. Significant findings are marked in bold.

**Table 2:** Main signs for ITB versus CD differentiation in cross-sectional imaging.

Findings	ITB	CD
**Site particularities**	Caecum retraction Patulous ileocecal valve Narrowed terminal ileum	Left colon
**Segments involved**	Shorter, fewer than 4	Longer, multiple
**Ulcers**	Transverse	Aphthous, longitudinal, deep
**Skip lesions**	Absent	Present
**Cobblestone appearance**	Absent	Present
Strictures	Short	Long
Wall thickening	Homogeneous	Asymmetrical
Stratification	Lost	Retained, intervening fat layer
Mucosal enhancement	Homogeneous	May be present
**Vasculature abnormalities**	-	Comb sign
		Ileal vasculature “jejunization”
**Lymph nodes**	Necrotic, conglomerated, large, peripheral enhanced	Smaller
**Extraintestinal signs**	Ascites, omental disease	Fibrofatty proliferation
**Pulmonary abnormalities**	Up to 25%	No

ITB = intestinal tuberculosis, CD = Crohn’s disease. Significant findings are marked in bold.

**Table 3:** Main pathological findings in the differentiation between ITB and CD [3,4,94,95,104].

	ITB	CD
Ulcers lined by histiocytes	++	-
with lymphoid cuffing	++	-
Granulomas large	++	-
multiple	++	-
confluent	++	-
foreign bodies giant	-	++
microgranuloma	-	++
Caseating necrosis	++	-
Giant cells	+	-

## Data Availability

No new data were created or analyzed in this study.

## Supplementary Materials

The following supporting information can be downloaded at: https://www.mdpi.com/article/10.3390/medicina62040794/s1, Supplementary File S1: Table original studies.docx; Supplementary File S2: Bayesian meta-analysis principles.docx.

## Abbreviations

The following abbreviations are used in this manuscript:

AFB	Acid-fast bacilli
ASCA	Anti-Saccharomyces cerevisiae antibodies
ATT	Antitubercular therapy
AUC	Area under the ROC curve
BMI	Body mass index
CD	Crohn’s disease
CEUS	Contrast-enhanced ultrasound
CNN	Convolutional neural network
CFP-10	Culture filtrate protein-10
ESAT-6	Early secreted antigenic target 6 kDa
EUS	Endoscopic ultrasound
G-CSF	Granulocyte-colony stimulating factor
HE	Hematoxylin and eosin stain
IBD	Inflammatory bowel disease
IFN	Interferon
IGRA	Interferon-gamma release assay
IL	Interleukin
ITB	Intestinal tuberculosis
LASSO	Least absolute shrinkage and selection operator
NAAT	Nucleic acid amplification test
NPV	Negative predictive value
PPAR	Peroxisome proliferator-activated receptor
PPD	Negative predictive value
PPV	Positive predictive value
TB	Tuberculosis
TNF	Tumoral necrosis factor
