# Supplementary material for: The Overlap Between Crohn’s Disease and Intestinal Tuberculosis: A Never-Ending Story
Source: Medicina (Kaunas). 2026 Apr 21;62(4):794. doi: 10.3390/medicina62040794 (PMC13117682; doi:10.3390/medicina62040794)
Supplement: Supplementary file 1 [file medicina-62-00794-s001.zip › medicina-4226975-supplementary/Supplementary File S1 Table original studies.pdf]

Supplementary Table S1. Characteristics for original studies analyzing CD and ITB. 1

Author, Year	Study period	Country	Patients	Features	Design
Jin et al., [4]	1996-2007	Korea	55 ITB - 42 CD	Pathology, fast-acid bacilli. PCR	Retrospective
Shi et al., [10]	2000-2015	China	85 ITB	Clinical, laboratory, pathology, and IGRA	Retrospective
Bannerjee et al. [15]	2020-2022	India	364 ITB-992 CD	Prevalence. Clinical features	Retrospective
Dhoble et al. [16]	2005-2019	India	224 ITB-348 CD	Prevalence	Retrospective
Chung et al. [17]	1998-2002	Korea	54 IT-65 CD	Clinical features, colonoscopy	Retrospective
Rolo et al. [20]	2008	Portugal	2 ITB-55 CD	Reclassification (pathology, bacteriology)	Retrospective
Sato et al. [21]	2004-2017	Japan	10 ITB	Reclassification (pathology, bacteriology)	Retrospective
Agranoff et al. [28]	Not stated	Uganda, Gambia	8 ITB – 30 IBD	Serum proteomics (serum amyloid A, transthyretin, neopterin, and CRP.	Retrospective
Ning et al. {29}	2016-2019	China	10 ITB-10 CD	Serum proteomics	Prospective
Zhang et al. [31]	2011-2014	China	21 ITB-30 CD	Serum proteomics	Prospective
Rukmangadachar et al. [32]	Not stated	India	5 ITB-5 CD	Serum proteomics	Prospective
Ma et al. [33]	2019-2020	China	48 ITB-68 CD	5 Serum metabolites	Retrospective
Ahuja et al. [34]	2008-2009	India	5 ITB-4 CD	Genetic-immune profile	Prospective
Rampal et al. [35]	2016-2017	India	47 ITB-23 CD	Immune profile	Prospective
Roy et al. [36]	2017-2020	India	27 ITB-25 CD	PCR, cytokine profile	Prospective
Tiwari et al. [37]	Not stated	India	21 ITB-32 CD	Cytokine profile	Prospective
Yoo et al. [38]	2012-2019	Korea	5 ITB-5 CD	Immune profile	Prospective
Yu et al. [39]	2008-2017	China	133 ITB-128 CD	Immune profile	Prospective
Gupta et al. [40]	Not stated	India	9 ITB-11 CD	Clinical, endoscopic features. Immune profile	Prospective
Jiang et al. [42]	2015-2019	China	9 ITB-103 CD	Serum antibodies	Retrospective
Pugazhendhi et al. [43]	Not stated	India	15 ITB-12 CD	Immune profile	Prospective
He et al. [45]	Not stated	China	6 ITB-16 CD	Microbiota profile	Prospective
Bajaj et al. [46]	Not stated	India	20 ITB-20 CD	Microbiota profile	Prospective
Khan et al. [47]	Not stated	India	32 ITB-69 CD	Microbiota profile	Prospective
Zeng et al. [48]	2008-2021	China	46 ITB	Clinical, laboratory, bacteriology, and imaging features	Retrospective
Lu et al. [50]	2015-2020	China	10 ITB	Clinical and endoscopic features	Retrospective
Amarapurkar et al. [51]	2002-2004	India	26 ITB-26 CD	Clinical, endoscopic, pathology, and serology features	Retrospective
Liu et al. [55]	2007-2012	China	30 ITB-38 CD	Clinical, endoscopic, pathology, and cross-sectional imaging features	Retrospective
Larsson et al. [56]	2009-2012	India	38 ITB-37 CD	Clinical, endoscopic, and pathology features	Prospective
Pulimood et al. [63]	1986-1996	India	20 ITB-20 CD	Pathology features	Retrospective
Cheng et al. [64]	2011-2012	China	69 ITB-107 CD	Clinical, endoscopic, laboratory, imaging, pathological, and serology features	Retrospective
Kim et al. [70]	2003-2015	Korea	16 ITB-19CD	Clinical and videocapsule endoscopy features	Retrospective
Park et al. [73]	2006-2011	Korea	17 ITB-64 CD	Cross-sectional imaging	Retrospective
Choi et al. []75]	2003-2014	China	84 ITB-116 CD	Clinical, colonoscopic, pathological, and ultrasonographic features	Retrospective
Israrahmed et al. [79]	2016-2018	India	61 ITB-24 CD	Cross-sectional imaging	Retrospective
Kim et al. [80]	2001-2010	Korea	18 ITB-21 CD	Clinical, cross-sectional features	Retrospective
Huang et al. [81]	2020-2021	China	51 ITB-182 CD	Cross-sectional imaging	Retrospective
Makanjuola et al. [82]	Not stated	Saudi Arabia	18 ITB-9 CD	Cross-sectional imaging	Retrospective
Kedia et al. [83]	2016-2018	India	65 ITB-23 CD	Cross-sectional imaging	Retrospective
Qiu et al. [87]	2008-2015	China	39 ITB-272 CD	Endoscopic ultrasound	Retrospective
Seth et al. [88]	2019-2020	India	11 ITB-6 CD	Cross-sectional imaging	Prospective
Ma et al. [89]	2015-2019	China	20 ITB	Cross-sectional imaging	Retrospective
Ko et al. [90]	2005-2008	Korea	25 ITB-25 CD	Cross-sectional imaging	Retrospective

Yahav et al. [91]	2012-2014	India	33 ITB-42 CD	Cross-sectional imaging	Retrospective
Seetharaman et al. [93]	2019-2020	India	22 ITB-12 CD	Cross-sectional imaging	Prospective
Ye et al. [96]	2010-2013	China	16 ITB-52 CD	Pathology features	Retrospective
Pulimood et al. [97]	1996-2000	India	33 ITB-30 CD	Pathology features	Retrospective
Han et al. [99]	2015-2023	China	2 ITB-93 CD	Pathology features (perianal disease)	Retrospective
Kirsch et al. [104]	1984-2004	Canada	18 ITB-25 CD	Pathology features	Retrospective
Watermeyer et al. [105]	Not stated	South Africa	50 ITB-46 CD	Pathology features	Retrospective
Bannerjee et al. [106]	2010-2011	India	8 ITB-9 CD	Pathology features	Retrospective
Das et al. [107]	Not stated	India	29 ITB-50 CD	Pathology features	Retrospective
Ince et al. [109]	Not stated	Turkey	24 ITB-28 ITB	Pathology features	Retrospective
Zhang et al. [110]	Not stated	China	18 ITB-56 CD	Pathology features	Retrospective
Kim et al. [111]	2007-2008	Korea	64 ITB-64 CD	IGRA, TST	Retrospective
Lei et al. [112]	2003-2011	China	88 ITB-103 CD	IGRA	Retrospective
Zhao et al. [113]	2015-2018	China	35 ITB-272 CD	IGRA	Retrospective
Sachdeva et al. [114]	2014-2021	India	59 ITB-49 CD	IGRA	Retrospective
Li et al. [118]	2008-2010	China	19 ITB-65 CD	Clinical, endoscopic, pathological, cross-sectional, and IGRA features	Retrospective
Fee et al. [120]	Not stated	USA	78 ITB	Clinical features	Retrospective
Makharia et al. [122]	Not stated	India	30 ITB-59 CD	Serology	Prospective
Zhang et al. [123]	Not stated	China	57 ITB-171 CD	Serology	Prospective
Kim et al. [124]	2007-2008	Korea	75 ITB-72 CD	Serology	Prospective
Kashima et al. [125]	Not stated	Japan	27 ITB-16 CD	Serology	Prospective
Patel et al. [126]	2011-2013	India	69 ITB	Clinical, laboratory, endoscopic, pathological, bacteriological, and PCR features	Retrospective
Gan et al. [128]	Not stated	China	36 ITB-26 CD	PCR. Fast-acid bacilli	Retrospective
Bellam et al. [130]	2018-2019	India	25 ITB-10 CD	Clinical, endoscopy, cross-sectional imaging, pathological features, PCR	Retrospective
Fei et al. [131]	2016-2018	China	42 ITB-46 CD	Pathological, bacteriology features. PCR	Retrospective
Kumar et al. [132]	2013-2014	India	37 ITB-43 CD	PCR	Prospective
Paulose et al. [135]	2016-2018	India	35 ITB	Clinical, endoscopic, and pathological features	Retrospective
Fei et al. [136]	2010-2013	China	29 ITB-36 CD	PCR	Retrospective
Ramadass [137]	2005-2006	India	24 ITB-44 CD	PCR	Prospective
Ye et al. [142]	2020-2023	China	66 ITB-34 CD	PCR	Prospective
Liu et al. [144]	2008-2021	China	441 CD	Prophylactic ATT	Retrospective
Aggarwal et al. [172]	2004-2015	India	106 ITB	ATT	Ambispective
Mao et al. [181]	Not stated	China	58 ITB-107 CD	Cross-sectional imaging	Prospective
Li et al. [185]	2003-2009	China	122 ITB-130 CD	Clinical and endoscopic features	Retrospective
Limsrivilai et al. [192]	2000-2018	Thailand. Hong Kong	147 ITB-383 CD	Clinical, endoscopic, laboratory, imaging, pathological, IGRA, PCR, and serology features	Retrospective
