# Supplementary material for: The Overlap Between Crohn’s Disease and Intestinal Tuberculosis: A Never-Ending Story
Source: Medicina (Kaunas). 2026 Apr 21;62(4):794. doi: 10.3390/medicina62040794 (PMC13117682; doi:10.3390/medicina62040794)
Supplement: Supplementary file 1 [file medicina-62-00794-s001.zip › medicina-4226975-supplementary/Supplementary File S2 Bayesian meta-analysis principles.pdf]

Bayesian meta-analytic models use a hierarchical framework similar to conventional random-effects models, but with prior distributions for the overall effect size and between-study heterogeneity, which can better estimate heterogeneous effects, handle small samples, and provide direct probability statements about outcomes. It permits a direct comparison between competing models and therefore improves estimation in diseases with relatively low frequency. In the Limsrivilai study, three models were constructed for differentiating between intestinal tuberculosis and Crohn disease, Model 1 includes the significant parameters with low heterogeneity ($I^2<50$) and likelihood ratio (LR) ≥2, Model 2 includes the significant parameters with low heterogeneity ($I^2<50$), and Model 3 includes the significant parameters, regardless of heterogeneity and LR. The best AUC was obtained for models 2 (0.920) and 3 (0.921).

Reference:

1. Limsrivilai J, Shreiner AB, Pongpaibul A, Laohapand C, Boonanuwat R, Pausawasdi N, Pongprasobchai S, Manatsathit S, Higgins PD. Meta-Analytic Bayesian Model For Differentiating Intestinal Tuberculosis from Crohn's Disease. Am J Gastroenterol. 2017;112(3):415-427. doi: 10.1038/ajg.2016.529.
